# Warming and neighbor removal affect white spruce seedling growth differently above and below treeline

**DOI:** 10.1186/s40064-015-0833-x

**Published:** 2015-02-13

**Authors:** Kyoko Okano, M Syndonia Bret-Harte

**Affiliations:** Institute of Arctic Biology, University of Alaska, Fairbanks, Fairbanks, AK, USA

**Keywords:** *Picea glauca*, Boreal forest, Climate change, Competition, Subarctic, Alaska

## Abstract

Climate change is expected to be pronounced towards higher latitudes and altitudes. Warming triggers treeline and vegetation shifts, which may aggravate interspecific competition and affect biodiversity. This research tested the effects of a warming climate, habitat type, and neighboring plant competition on the establishment and growth of white spruce (*Picea glauca* (Moench) Voss) seedlings in a subarctic mountain region. *P. glauca* seedlings were planted in June 2010 under 4 different treatments (high/control temperatures, with/without competition) in 3 habitats (alpine ridge above treeline/tundra near treeline /forest below treeline habitats). After two growing seasons in 2011, growth, photosynthesis and foliar C and N data were obtained from a total of 156, one-and-a-half year old seedlings that had survived. Elevated temperatures increased growth and photosynthetic rates above and near treeline, but decreased them below treeline. Competition was increased by elevated temperatures in all habitat types. Our results suggest that increasing temperatures will have positive effects on the growth of *P. glauca* seedlings at the locations where *P. glauca* is expected to expand its habitat, but increasing temperatures may have negative effects on seedlings growing in mature forests. Due to interspecific competition, possibly belowground competition, the upslope expansion of treelines may not be as fast in the future as it was the last fifty years.

**Electronic supplementary material:**

The online version of this article (doi:10.1186/s40064-015-0833-x) contains supplementary material, which is available to authorized users.

## Introduction

Annual average temperature in Alaska has increased 1.9°C over the past 50 years and is projected to rise from 2.8°C to 7.2°C above the 1960s-1970s baseline by the end of this century (Karl et al. 2009). The projection is higher than the estimated 2.4°C to 6.4°C rise in global average temperature over the same period of time (IPCC 2008). This recent climate warming is expected to alter the relative abundance of arctic and subarctic vegetation in Alaska. Photographic comparisons of past and current vegetation (Sturm et al. 2001, Tape et al. 2006, Hamm 2007, Stueve et al. 2011) have shown both northward and altitudinal expansions of shrubs and treeline into tundra. Studies in Denali National Park and Preserve (DNPP) reported the maximum treeline advance was 150 m upslope into tundra (Stueve et al. 2011) and the average treeline advance was 40 to 60 m (Hamm 2007) in the last 50 years. Those studies suggest that higher temperatures stimulate successful recruitment, and possibly growth of treeline species, predominantly *Picea glauca* (white spruce) in the subarctic mountain region.

*P. glauca* is also one of the dominant species in boreal forests; however, higher temperatures that have been observed more frequently since 1950 are linked to drought stress, resulting in decreased growth rates of *P. glauca* (Wilmking et al. 2004). Because of desiccation, mature trees could become more susceptible to insect outbreaks, diseases and wildfires, thus increasing mortality. Models project that the growth rate of *P. glauca* could decrease to 0 - 20% of the long-term mean by 2100 due to drought stress, which might lead to species elimination (Juday et al. 2005). Recent studies of marginal tree population have shown both positive and negative responses to climate warming (e.g. Wilmking et al. 2004). Experimentation is necessary to uncover the mechanistic processes at work.

In addition to altering the growth of adult trees, climate change may have strong effects on sexual reproduction and the establishment of seedlings, which are the most vulnerable life stages of a tree. Warm temperatures are thought to be important for the establishment of seedlings and their survival during the first 50 years of growth (Szeicz and MacDonald 1995). However, rather than temperature effect itself, the growth of *P. glauca* seedlings in subarctic alpine forests on south-facing slopes was limited by winter drought and cold-induced photoinhibition in the early spring and/or the early morning in the summer (Danby and Hik 2007, Slot et al. 2005). Further, even if warming positively affects seedlings, drought stress could also be increased. Also, the viability of seeds from trees growing at higher elevations is usually lower than from those growing at lower elevations (Roland et al. 2013), which may inhibit upslope expansion.

Seedlings of *P. glauca* at a higher elevation need to acclimate to harsh new abiotic conditions, and interact with other plant species that are already present. Positive interactions with neighboring plants are crucial for small spruce seedlings in harsh environments. Choler et al. (2001) found that the effects of neighboring plants are facilitative rather than competitive in high and exposed sites. However, Bret-Harte et al. (2008) reported that some arctic species responded positively to neighbor removal while others did not. The response of *P. glauca* seedlings to neighboring plants under a changing climate is thus, likely to be determined by complex factors that are not fully understood.

The goal of this study was to understand the mechanistic effects of warming and interspecific competition on the performance of seedlings of *P. glauca* in a range of habitats above and below the current treeline on subarctic Alaskan mountains, in order to inform future conservation. Our hypotheses are as follows. 1) Growth of *P. glauca* seedlings above treeline is reduced relative to seedlings below treeline due to negative environmental conditions such as low temperatures and low water content in soil. 2) Warming in a cold environment positively affects seedlings as long as no drought stress occurs. 3) Interactions between *P. glauca* and neighboring plants are mostly competitive, but harsh conditions can trigger less competitive or even facilitative effects. We planted *P. glauca* seedlings at three different habitats to test habitat effects (hypothesis 1). At each site, the seedlings were planted inside or outside a small greenhouse to compare the effects of warming vs. ambient temperatures (hypothesis 2). Both inside and outside the greenhouses, the seedlings were planted either with neighboring plants or with neighbors removed, to examine the interspecific interaction at ambient and raised temperatures (hypothesis 3). Our results will provide insight how seedlings of treeline species will respond to climate change, and how habitat type and interspecific competition affect treeline shifts and the establishment and growth of spruce seedlings.

## Results

### Environmental manipulation

Mean ambient air temperatures differed by 2°C - 3.5°C among three habitat types during the summer growing season in 2010 (Table 1). Because of a failure of a logger below treeline, air temperature data in 2011 were obtained only from the above and near treeline sites, whose difference was only 1°C. Greenhouses increased air temperatures by 3.22°C - 6.22°C in 2010 and 2.84°C - 3.37°C in 2011. Soil temperatures at 5 cm from the surface were only obtained below treeline in 2011, where mean temperature in the greenhouse was raised by 5.18°C. In 2012, mean soil temperatures inside vs. outside the greenhouses at the above and the below treeline sites differed less than 1°C while inside the greenhouse was 6.72°C higher than outside at the near treeline site.

**Table 1:** **Comparisons of environment types at the study sites**

**Site**		**Below treeline**	**Near treeline**	**Above treeline**
Elevation (m)		618	670	1169
Vegetation type		Spruce forest	Shrub tundra	Alpine tundra
Two dominant species (or growth forms)		Moss	Moss	*Dryas*
		*Empetrum*	Lichens	Graminoids
Summer 2011				
Air temperature mean (°C)	Ambient	n/a	10.36	9.27
	Inside greenhouse	n/a	13.73	12.11
Mean of daily maximum	Ambient	22.4*	18.60	15.03
	Inside greenhouse	31.39*	25.41	21.01
Mean of daily minimum	Ambient	6.07*	4.25	4.94
	Inside greenhouse	7.13*	5.63	6.20
Soil temperature mean (°C)	Ambient	3.42	n/a	n/a
	Inside greenhouse	8.60	n/a	n/a
2012 Soil temperature mean (°C)	Ambient	9.75	3.69	8.91
	Inside greenhouse	10.00	10.41	8.94
2010 Air temperature mean (°C)	Ambient	12.51	10.69	8.73
	Inside greenhouse	15.73	16.91	12.39
January 2011 Air temperature mean (°C)		−15.60	−13.13	−9.51
Light (μmol m-2 s-1) Sunny day	Ambient*	1656.50	n/a	1276.57
	Inside greenhouse*	1241.52	n/a	855.70
Light (μmol m-2 s-1) Cloudy day	Ambient*	376.86	512.17	500.58
	Inside greenhouse*	164.85	318.85	382.99
Air % Relative humidity (%)	Ambient	n/a	93.56	86.88
	Inside greenhouse	n/a	72.19	80.21
2012 Air Relative humidity (%)	Ambient	78.23	84.61	81.20
	Inside greenhouse	73.17	67.12	71.91
Soil % Volumetric water content	Ambient*	n/a	47.15	23.60
	Inside greenhouse*	n/a	35.00	19.50
Soil % Volumetric water content (Sampling)		53.42	52.49	20.36
Soil CN ratio		17.72	8.63	13.26
Soil texture		Sandy Loam	Loam	Sandy Clay Loam
Soil Bulk density (g/cm3)		0.182	0.795	0.559
Soil pH		6.0	5.0	6.5
Depth of thaw (cm)*		74.83	72.71	45.00

*Data 2010.

Three habitat types: above, near and below treeline sites. Data were obtained during a growing season (except January temperatures). Mean summer temperatures are recorded between 12-Jun-12-2011 and 29-Aug-2011 (the below treeline site was measured in 2010).

Light response curve (Additional file 1: Figure S1) showed that seedlings’ photosynthetic performance reached its maximum a little above 500 μmol m^−2^ s^−1^ light intensity, indicating that our measuring light intensity of 1500 μmol m^−2^ s^−1^ was beyond seedlings’ light saturation at all sites.

Greenhouses blocked summer precipitation, which decreased air relative humidity (RH) and soil percent volumetric water content inside the greenhouse to 77.2% - 92.3% (similarly in 2012) and 74.2% - 82.6% of ambient levels, respectively (Table 1). The greenhouses also decreased light intensity on average to 68% (±2.78 SE) of ambient light levels depending on weather conditions; however, this reduction of light intensities was not statistically different (t-test, P = 0.45). The inside greenhouse light intensity had reached over 1000 μmol m^−2^ s^−1^ (Table 1) at the below treeline site where the most shaded condition was expected among the sites. Light intensity in the greenhouses was always above the levels that caused photosynthetic light saturation (a little above 500 μmol m^−2^ s^−1^; Additional file 1: Figure S1) on sunny days. This suggested that seedlings inside the greenhouses at all sites were not shade-adapted. On cloudy days, light levels in greenhouses were below saturation (Table 1). However, all photosynthetic measurements were made at saturation (1500 μmol m^−2^ s^−1^). Although caution is needed in interpreting data, we believe that the use of the greenhouses did not affect our photosynthesis results.

### Seedling size

For several variables, results of three-way ANOVA indicated that there were significant interactions between habitat type and temperature, and between temperature and removal treatments (Table 2). High temperatures influenced the growth response (relative growth rate, RGR) of both height and number of needles of *P. glauca*, but not in the same way at each habitat. Above treeline, both height and number of needles were increased significantly by elevated temperatures. Near treeline, height was increased with marginal significance by high temperatures, but the number of needles was decreased, although the differences between high and ambient temperature treatments were not significant. Below treeline, height and number of needles were decreased when temperatures were high, but not significantly.

**Table 2:** **Results of statistics (general linear model, GLM)**

**Factor**														
	**Habitat type (H)**		**Temperature (T)**		**Removal (R)**		H*T		H*R		T*R		H*T*R	
	**F**	**P**	**F**	**P**	**F**	**P**	**F**	**P**	**F**	**P**	**F**	**P**	**F**	**P**
Size measurements	Ndf: 2	Ddf:144	Ndf: 1	Ddf:144	Ndf: 1	Ddf:144	Ndf: 2	Ddf:144	Ndf: 2	Ddf:144	Ndf: 1	Ddf:144	Ndf: 2	Ddf:144
(1) RGR Height^†^	9.51	**0.0001**	11.26	**0.001**	0.23	0.633	15.43	**<0.0001**	1.96	0.145	5.05	**0.026**	1.43	0.244
(2) RGR # of needles^†^	28.14	**<0.0001**	1.79	0.183	9.22	**0.003**	3.70	**0.027**	1.47	0.232	0.50	0.480	0.71	0.493
(3) RGR Needle length^‡^	7.10	**0.0011**	0.19	0.664	0.61	0.438	1.72	0.182	0.18	0.837	0.06	0.807	0.97	0.380
ᅟ														
Photosynthesis	Ndf: 2	Ddf:143	Ndf: 1	Ddf:143	Ndf: 1	Ddf:143	Ndf: 2	Ddf:143	Ndf: 2	Ddf:143	Ndf: 1	Ddf:143	Ndf: 2	Ddf:143
(4) Photosynthesis*	32.43	**<0.0001**	0.01	0.9312	0.44	0.510	52.92	**<0.0001**	1.24	0.2916	4.22	**0.042**	0.65	0.523
ᅟ														
% N and ^13^C	Ndf: 2	Ddf:47	Ndf: 1	Ddf:47	Ndf: 1	Ddf:47	Ndf: 2	Ddf:47	Ndf: 2	Ddf:47	Ndf: 1	Ddf:47	Ndf: 2	Ddf:47
(5) %N**	5.80	**<0.0001**	0.12	0.735	7.19	**0.010**	1.91	0.160	1.55	0.223	0.05	0.826	1.13	0.333
(6)^13^C	8.01	**0.001**	10.27	**0.002**	0.39	0.536	4.80	**0.013**	0.44	0.646	0.85	0.361	0.17	0.200
ᅟ														
ANCOVA	Ndf: 2	Ddf:46	Ndf: 1	Ddf:46	Ndf: 1	Ddf:46	Ndf: 2	Ddf:46	Ndf: 2	Ddf:46	Ndf: 1	Ddf:46	Ndf: 2	Ddf:46
(7) %N**	5.95	**0.0051**	0.38	0.543	0.13	0.719	37.58	**<0.0001**	1.15	0.324	0.44	0.512	0.39	0.680
(8)^13^C	16.57	**<0.0001**	0.60	0.441	0.50	0.481	36.87	**<0.0001**	0.91	0.409	0.57	0.456	0.19	0.832

^†^rank, ^‡^yt = y/s, *log(y + 1), and **log (y) transformation to achieve homogeneity of variance. (Bold: P < 0.05).

Three-way ANOVA for relative growth rates (RGR) in (1) height, (2) number of needles, (3) length of the longest needle for size measurements; (4) photosynthetic rate, (5)%N and (6) δ^13^C. ANCOVA for photosynthetic rate with (7)%N and (8) δ^13^C. The interactions between the treatments were shown; e.g., H*T (habitat type and temperature). Significant differences were bold. Data were collected from 156 seedlings (one failed for photosynthesis, n = 155). ANCOVA was run using a subset of seedlings (n = 59). Transformation methods were suggested by Dr. J. McIntyre of Department of Mathematics and Statistics, UAF.

**Figure 1:**
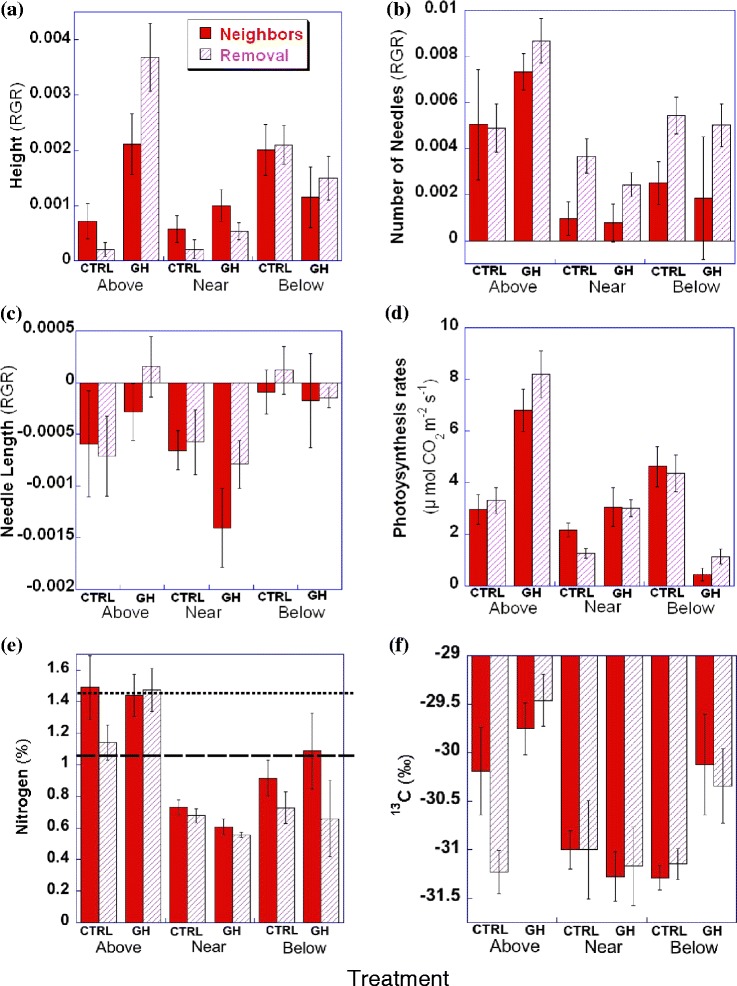
**Results of growth response of** ***Picea glauca*** **.** Relative growth rates (RGR) of **(a)** height, **(b)** number of needles, and **(c)** length of the longest needle (n = 156). **(d)** Gross photosynthesis rate (n = 155). **(e)** Percentage of N content (n = 59). The upper dotted line is an adequate nitrogen level (1.45%). The lower dotted line is a severe deficiency level (1.05%). **(f)** δ^13^C content (n = 59). CTRL = control temperatures. GH = elevated temperatures. Above = above treeline site, Near = near treeline site, Below = below treeline site. Error bars show one standard error.

Seedlings in higher temperatures increased heights significantly above treeline (F _(1, 51)_ = 40.17, P < 0.0001, one-way ANOVA) and marginally significantly near treeline (F _(1, 54)_ = 3.06, P = 0.086, one-way ANOVA). Although the difference below treeline was not significant (F _(1, 45)_ = 2.00, P = 0.16, one-way ANOVA), warmed seedlings were 30% shorter without neighbors and 45% shorter when neighbors were present relative to seedlings in the ambient temperature. There was a significant interaction between temperature and removal for RGR in height because there were increased competition effects overall when the temperature was higher (Table 2, Figure 1a).

Neighbor removal increased number of needles at all sites but one (control above treeline, insignificant difference) and there was no significant interaction with temperature (Table 2, Figure 1b). The number of needles increased with higher temperatures above treeline (F _(1, 51)_ = 6.97, P = 0.01, one-way ANOVA), but there were no significant effects at the other two sites. Length of the longest needle decreased over the summer due to leaf turnover. This decrease in leaf length was significant only for those at the near treeline site (P = 0.001, Table 2, Figure 1c).

Overall, warming affected the RGR of seedlings positively above treeline, but had slightly positive to weakly negative effects at the lower two habitat types.

### Photosynthetic response

There was a significant interaction between temperature and habitat type (P < 0.0001, Table 2), because elevated temperatures significantly increased photosynthetic capacity (photosynthetic rate at 1500 μmol m^−2^ s^−1^) above (F _(1, 51)_ = 33.93, P < 0.0001, one-way ANOVA) and near (F _(1, 54)_ = 10.80, P = 0.0018, one-way ANOVA) treeline, but significantly decreased photosynthetic capacity below treeline (F _(1, 45)_ = 49.82, P < 0.0001, one-way ANOVA). Although post hoc Tukey HSD tests were not significant, the interaction between temperature and removal effects were significant (F _(1, 143)_ = 4.22, P = 0.042), because competition at the near treeline site became marginally more pronounced under elevated temperatures than under ambient temperatures (F _(1, 54)_ = 3.88, P = 0.054, two-way ANOVA, Figure 1d).

### Foliar N and C, and their relationships with photosynthesis

Foliar N and C were indicators of seedlings’ nitrogen availability and drought stress, respectively. Nitrogen concentration of needles was significantly higher above treeline where photosynthetic rates were also high (Table 2, Figure 1e). There, the foliar %N was at a level previously suggested to be adequate for growth (1.45%, Carter 1992). All of the seedlings near treeline and most of the ones below treeline had nitrogen levels indicative of severe deficiency 1.05%, (Carter 1992). Except in one treatment, seedlings with neighbors had significantly higher nitrogen concentration in needles than did ones without neighbors at the same site (Table 2, Figure 1e). δ^15^N values of *P. glauca* have been observed to range between −1‰ and −11‰ due to variations in nitrogen availability to plants and their relationships with mycorrhizal fungi that supply organic nitrogen; hence, %N and δ^15^N are often correlated (Hobbie et al. 2009). In our study, all but one (−0.80‰) of the 59 subsampled seedlings had a positive value of δ^15^N (+2.91‰ ± 0.19SE, data not shown), and %N did not correlate with δ^15^N (Additional file 1: Figure S2).

With respect to C, δ^13^C levels in needles of seedlings growing above treeline were less negative (more enriched) than the levels in needles from other habitat types (Figure 1f). Overall, δ^13^C levels under ambient temperatures were more negative (more depleted) than the ones under elevated temperatures. This could indicate a small amount of water stress in the greenhouse. However, this difference was only significant above treeline. All seedlings had δ^13^C values of −29.42‰ ~ −32.81‰, which is within the range or even slightly more negative than previously published data for *Picea* (−26‰ to −31‰; Grossnickle 2000, or −29‰ for *P. glauca*; Livingston et al. 1999). This indicates that no seedlings suffered from severe water stress, that they could open their stomata in order to obtain CO_2_, and could discriminate against heavy ^13^C during needle production.

In order to assess whether foliar %N or δ^13^C explained the differences in photosynthetic performance, we ran ANCOVAs, using photosynthesis data as a dependent variable and either %N or δ^13^C as covariates. In both cases, the covariates eliminated the interaction effects between temperature and removal (Table 2), but not the habitat by temperature interactions and the main effects of habitat type. Variation in %N or δ^13^C partially explained the significant effect of habitat on photosynthetic rates, but did not make it insignificant in the ANCOVA, suggesting that additional factors that differ among habitats were important.

## Discussion

Elevated temperature increased growth and photosynthetic rates most strongly above treeline and slightly near treeline, but decreased them below treeline, which indicates that warmer temperatures have different effects depending on habitat. Climate warming can cause drought stress on plants, and such unfavorable abiotic conditions commonly trigger facilitative effects in a plant community. However, our δ^13^C results suggest that temperature-induced drought stress was not an important factor in our experiment, and we saw no evidence for facilitation, suggesting that climate warming will have a positive effect on the growth of seedlings where it is currently too cold for *P. glauca*. Warmer temperatures increased competition between spruce seedlings and neighboring plants at all sites.

Above treeline, where seedlings did not suffer from drought stress, photosynthetic rates were increased by elevated temperatures. The seedlings above treeline, both inside and outside the greenhouse, had foliar nitrogen levels close to what is considered an adequate level (1.45%, Carter 1992), yet the seedlings outside at ambient temperatures did not photosynthesize as much as the seedlings at high temperatures, indicating that higher temperatures increased photosynthesis rates when N was not limiting. Higher temperatures could increase either activity or production of Ribulose 1,5-bisphosphate carboxylase/oxygenase (Rubisco) and other Calvin cycle enzymes, which would amplify carboxylation capacity. Plants tend to produce more Rubisco than needed, particularly when nitrogen is abundant (Stitt and Schulze 1994). The high foliar nitrogen concentration suggests abundant Rubisco. Therefore, it appears that an increased amount of Rubisco allows the seedlings under high temperatures to fix more carbon and allocate enough photosynthates for both height and needle growth.

Seedlings near treeline also increased their photosynthetic rates significantly under warming relative to ambient temperatures at the same habitat type, although photosynthetic rates near treeline were the lowest of all sites. Unlike seedlings above treeline, warmed seedlings allocated more carbon to height growth than to needle production. In the ambient temperature, low photosynthetic rates at the near treeline site may have occurred because low soil temperatures induce stomatal closure and inhibit photosynthesis (Goldstein et al. 1985) and can suppress the height growth of *P. glauca* (Zhang and Dang 2007). Near treeline was not invaded by spruce until recently (Hamm 2007). Mineral soil just below the organic soil (a depth of 15–20 cm) was frozen at the end of May and soil quality differs from other sites (Table 1), which might affect soil turnover and nutrient availability, and subsequently establishment of spruce. The increased soil temperature caused by the greenhouse (Table 1) could increase the photosynthetic rates and promote seedlings’ growth. However, because these seedlings were nutrient-limited, there may have been a trade-off between above and belowground growth, which may have resulted in less carbon available for aboveground growth and needle production than at the above treeline site. Alternatively, allocation to height over needle production may have resulted from competition for light. This allocation pattern has been seen previously in *Picea asperata*; elevated temperatures significantly increased height and root mass ratio, but decreased leaf mass ratio for *P. asperata* seedlings (Yin et al. 2008). Perhaps a lack of needle production prevented the near treeline seedlings from a rapid increase in photosynthetic performance, and hence, from a rapid increase in growth. Still, recent warming temperatures have a potential to promote *P. glauca* seedlings by increasing photosynthesis and height growth near treeline.

Trade-offs are important for the survival of plants in infertile environments, which are common in the Arctic and subarctic. Instead of partitioning all photosynthates to tissues that support growth, slow growing species invest in storage and traits that conserve resources (Grime et al. 1997, Grime 2001, Díaz et al. 2004). Alternatively, plants may trade-off resource allocation between roots and shoots (Tilman 1988). Conifers, including spruce, tend to allocate photosynthates toward belowground biomass when either soil nutrients or water are scarce (Waring 1987, Grossnickle 2000). In some places, positive relationships between aboveground net primary productivity and belowground net primary productivity have been seen, but in boreal forests, there is often a negative relationship (Litton and Giardina 2008). In boreal forests, there is also a negative relationship between the mean annual temperature and total belowground carbon flux, presumably due to changes in resource allocations with increasing nutrient availability caused by rising temperatures (Litton and Giardina 2008). While our experiment focused on aboveground allocation only, as temperature increased, the seedlings showed differences in allocation by habitat type, indicating differences in their growth strategies. Seedlings above treeline increased total photosynthates, thereby increasing in height and the number of needles, perhaps also increasing in belowground biomass. Seedlings near treeline apparently allocated photosynthates mainly toward height and possibly some toward belowground structures, presumably due to lack of nutrient availability as indicated by their low foliar N (Waring 1987, Grossnickle 2000).

In contrast to the other two habitats, warming affected seedlings negatively below treeline; there, elevated temperature decreased photosynthetic capacity (Figure 1d). The below treeline site is a mature spruce forest whose current environment is favorable for mature *P. glauca* trees. However, if the current temperature regime is not the same as when these trees were established, and it is near the upper limit of the species’ tolerance now, a slight increase in temperature could make the habitat unfavorable to the species. During the 2010 growing season, both the average temperature and the average maximum temperature in a greenhouse below treeline were much higher, by 3°C and 9°C, respectively, than the ambient temperatures (Table 1). The optimal temperatures for photosynthesis of *P. glauca* seedlings have been reported as 15°C or 25°C (Man and Lieffers 1997). Mature trees of *P. glauca* in the Alaska Range decreased their growth when the average July temperature in Fairbanks reached 16°C or higher, which on average corresponds to 11.5°C at the Headquarter of DNPP (Wilmking et al. 2004). The average temperature in the greenhouse below treeline (15.7°C) was greater than this threshold (Wilmking et al. 2004), and the average maximum temperature (31.1°C) is also too high for the optimal temperature in photosynthetic performance of seedlings (Man and Lieffers 1997). Our results suggested that the current habitat of mature spruce forests will likely become less favorable to both mature and juvenile *P. glauca* species as climate warms in the future.

Although negative effects of warming on *P. glauca* have been reported by previous studies (e.g. Barber et al. 2000, Wilmking et al. 2004), considering the species’ distribution, our result that the environment of below treelines would become unfavorable to *P. glauca* due to elevated temperatures was surprising. *P. glauca* occurs from Alaska to South Dakota, Wyoming and northern New England, and the southern limits fall into the 18°C July temperature isotherm (Nienstaedt and Zasada 1990). Alaska is near the northern edge of its distribution and why a species that can reside in more southern areas has suffered from summer warming in Alaska is puzzling. One possible explanation is due to ecotypic genetic differentiation between Alaskan and non-Alaskan *P. glauca*. Alaskan *P. glauca* had a unique haplotype of chloroplast DNA that distinguished it from *P. glauca* in northeastern North America (Anderson et al. 2006, de Lafontaine et al. 2010). *P. glauca* in Alaska was assumed to have been able to survive the last glacial maximum in the Beringia refugium, not only due to the local microclimate, but also due to being adapted to severely cold climate (Anderson et al. 2006). Perhaps the seedlings in our experiment originating in DNPP are genetically better adapted to cold temperature more than *P. glauca* from different origins, and are more sensitive to warmer temperatures.

Our growth and photosynthesis results suggest that interactions between *P. glauca* and neighboring plants were mostly competitive, and that competition was aggravated under elevated temperatures. Seeing strong competitive effects above treeline contrasts with previous alpine studies that concluded facilitation was more important than competition between plants under a harsh abiotic environment (Callaway 1998, Cavieres et al. 2002). Limiting factors for the growth of plants at high altitudes include non-resource factors such as temperatures, winds and disturbance of soil rather than resources such as soil nutrients; the opposite is true at lower altitudinal sites (Callaway et al. 2002). At the two higher sites (above and near treeline), rising temperatures eliminated one of the non-resource limiting factors, so soil resource may have become a stronger limiting factor. On the other hand, the seedlings below the treeline site, the current habitat of a mature forest, recorded negative effects of elevated temperatures on the seedlings, but again no facilitation effects were seen. Where facilitation develops in harsh environments, there is likely a trade-off between the damaging effect of winter winds, which is lessened by being close to neighbors, and reduced nutrient availability due to uptake by neighbors. Because no physical damage by winds occurred in the mature forest, there was no obvious advantage there for seedlings to have neighboring plants. Seedlings below treeline were mostly under severe nitrogen deficiency, so belowground competition was presumably the limiting factor for the below treeline seedlings, as Callaway et al. (2002) suggested.

Our experiment suggested that competition and nutrient availability may limit growth of the *P. glauca* seedlings under warming even though warming has a positive effect. This result is consistent with previous *P. glauca* experiments. Warming increased the growth of transplanted *P. glauca* seedlings for more than three years in arctic tundra sites and belowground competition limited growth there, presumably due to nutrient competition (Hobbie and Chapin 1998). Effects of competition with *Populus tremuloides* (aspen) on the growth of *P. glauca* increased more than proportionally when temperatures rose (Cortini et al. 2012). *P. glauca* seedlings in boreal forests did not obtain any benefits from neighboring shrubs and herbaceous plants (Cortini and Comeau 2008). Sullivan et al. (in press) found evidence that the growth of *P. glauca* was increased by increased nutrient availability due to warmer soil temperatures, rather than air temperature. These results are similar to what we found and suggest that competition, especially belowground competition between spruce seedlings and neighboring plants, could limit the growth of *P. glauca* seedlings when temperatures increase in the future. Since our study focused on aboveground allocation, further studies on seedlings’ resource allocation including belowground allocation are needed to support this interpretation. If the negative effect of competition becomes more pronounced than the positive effect of warming, expansion of spruce habitat in the future may not occur so rapidly as treeline expansions have proceeded for the past 50 years in DNPP (Stueve et al. 2011, Hamm 2007).

Our study centered on seedlings of *P. glauca* at treeline ecotones, while many other studies have focused on mature trees of this species. Because successful recruitment and establishment of seedlings are essential for expansion of treeline, studying the response of seedlings to changing climate is necessary. Most importantly, seedlings are generally more vulnerable than mature trees and likely more sensitive to environmental changes such as warming temperatures. Successful recruitment years may be followed by decades with no recruitment at treeline as in Russia (Gervais and MacDonald 2000). Similar observations of pulses of recruitment have been made in Sweden (Kullman 1991, Dalen and Hofgaard 2005). This indicates that seedlings are sensitive to interannual variability in climate, which affects their recruitment, establishment, or mortality. However, trees might need only a few summers of extraordinarily good weather for recruitment to keep up the species’ population (Körner 2012). Our study focused on establishment and growth of surviving seedlings, and found that habitats above and near treeline have a potential to promote the growth of spruce seedlings as climate warms. Further studies of seed viability and germination, growth of older seedlings and saplings, and causes of mortality of seedlings in the field before and after establishment would also improve our understanding of seedlings’ growth capacity at treeline ecotones in the warmer future.

## Conclusion

Our results suggest that increasing temperatures would favor the growth of *P. glauca* seedlings at those places where it is too cold now. On the other hand, it is questionable whether Alaskan *P. glauca* would be able to continue to grow in its current habitat below treeline as climate warms. Current mature forests could become unfavorably warm for seedling growth of *P. glauca*. Warming may also promote increased competition, likely belowground, which could slow rates of habitat expansion of *P. glauca* above treeline. *P. glauca* is likely to remain the dominant treeline species, and treeline expansion will keep occurring in this subarctic mountain region as the climate warms, but may not be as fast as we have seen during the past 50 years.

## Materials and methods

### Study area description

The study area was located in Camp Denali and North Face Lodge, one of the private inholdings within DNPP near the southwestern end of the Kantishna Hills approximately 46.7 km north of the Alaska Range (Hamm 2007). The closest weather station is approximately 3 km away at Wonder Lake (63° 29’25”N, 150° 52’17”W, elevation 646 m, which is between our near treeline and below treeline sites. Western Regional Climate Center. http://www.wrcc.dri.edu) indicated that mean temperature, mean humidity, and total precipitation during the experimental period in 2011 (June 12 - August 29) were 10.75°C, 75.78% and 205.46 mm, respectively. The Wonder Lake temperature was similar to that measured in our near treeline site (Table 1). Three experimental sites were established, at different habitat types relative to the current treeline: a site above, near, and below (Table 1). At each site, two plots (on relatively flat ground no more than 5 m apart) were randomly chosen within vegetation representative of that where *P. glauca* occurred in the area (if it occurred).

**Figure 2:**
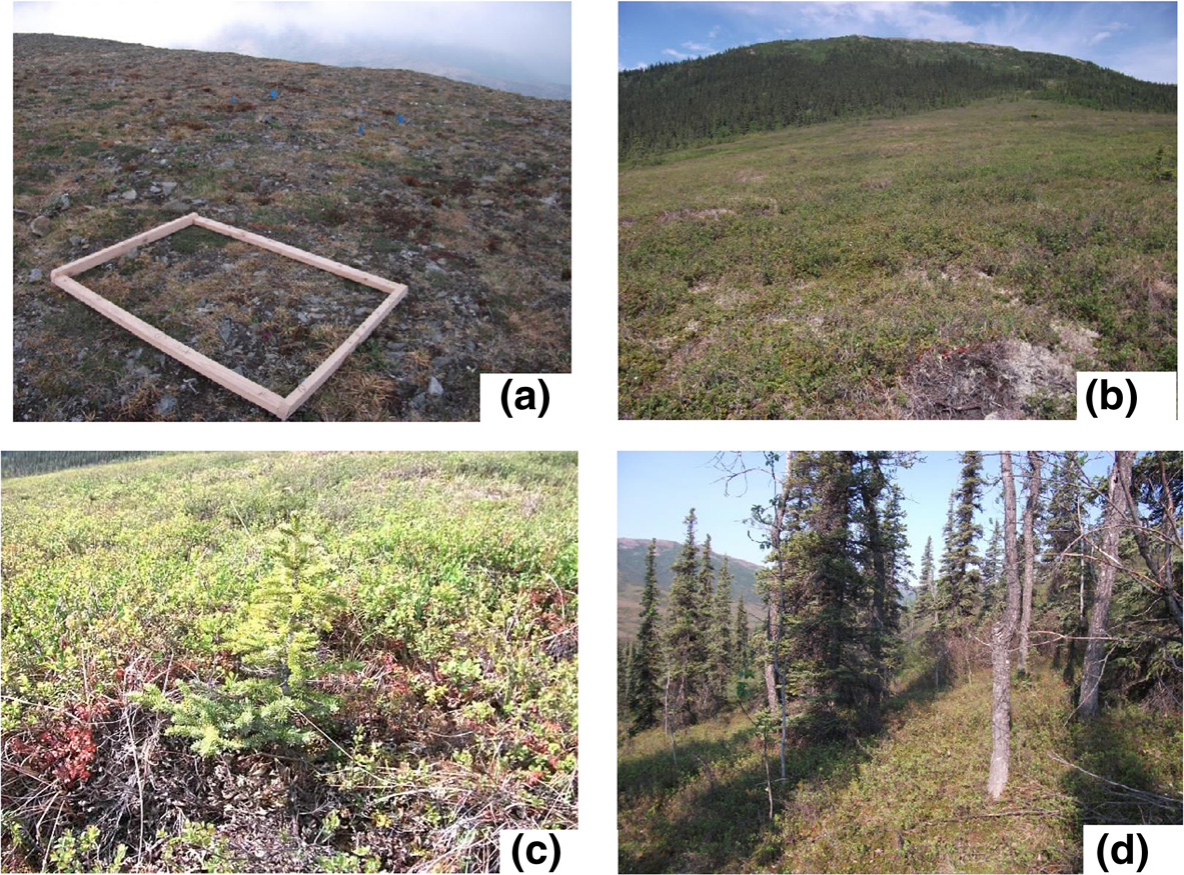
**Experimental sites. (a)** Above treeline. **(b)** Near treeline. **(c)** a sapling found at the near treeline site. **(d)** Below treeline.

The highest, above treeline site (63°32’17”N, 150°53’24”W, elevation 1169 m) was on an exposed hilltop with no aspect and no shade (Figure 2a). The area was covered with subarctic and alpine tundra species, but rocks and soil were also exposed. Vegetation of the study area included evergreen (*Cassiope tetragona*, *Loiseleuria procumbens*, *Rhododendron lapponicum*, *Dryas octopetala*) and deciduous (*Salix* spp*.*) tundra shrub species, forbs (*Primula cuneifolia*, *Anemone narcissiflora*, *Oxytropis nigrescens*), graminoids (*Carex* spp), and moss and lichen species.

The near treeline site (63°31’09”N, 150°53’52”W, elevation 670 m) was on a south-facing slope in open shrub tundra with occasional, scattered *P. glauca* saplings (Figure 2b, c). It was lower than altitudinal treelines elsewhere in the Alaska Range (up to 1,550 m, Sveinbjörnsson 2000), possibly because of poorly drained soils underlain by permafrost. Recent climate change, however, has promoted white spruce establishment and growth in this area for the past 50 years (Hamm, 2007). Treeline is defined not only by elevation (Sveinbjörnsson 2000); considering heterogeneity of treeline including topology, interaction with neighboring vegetation and local climate that affect treeline, we followed the broad definition of treeline as Stueve et al. (2011) employed, which made us define this site as “near treeline tundra.” There were very few juvenile *P. glauca* saplings in this area (Figure 2b,c). Those that could be measured had an average age between 18 and 19 years (n = 3), based on counting annual extension growth. Most of the area was open and vegetation was composed of evergreen (*Vaccinium vitis-idaea*, *Rhododendron subarcticum* (USDA, ARS, National Genetic Resources Program; formerly known as *Ledum palustre* ssp*. decumbens*), *Empetrum nigrum*) and deciduous (*Betula nana*, *Vaccinium uliginosum*, *Rubus chamaemorus*) shrub species, graminoids (*Carex* spp), lichens and mosses. The moss layer, mostly composed of *Sphagnum* spp., was between 5 cm and 10 cm deep, and was heavily mixed with lichens.

The below treeline site (63°32’10”N, 150°54’24”W, elevation 618 m) was located on a south-facing slope in a mature white spruce forest (Figure 2d). We cored four trees (randomly selected) near our site. The tree cores gave an average age of 127.4 years after correcting for time to grow to core height (Lloyd and Fastie 2003, Hamm 2007), although this may not represent age of the entire stand. The study site was not a closed canopy, but was sunny with occasional shading by mature trees (*P. glauca*, *Populus balsamifera*). Vegetation was composed of evergreen (*Juniperus communis*, *V. vitis-idaea*, *Rhododendron groenlandicum* (formerly known as *Ledum palustre ssp. groenlandicum*), *Empetrum nigrum*) and deciduous (*V. uliginosum*) shrub species, and a thick (more than 20 cm deep) layer of mosses.

### Seedling preparation

Seedlings of *P. glauca* were grown in a greenhouse at University of Alaska Fairbanks (UAF). Seeds in cones were harvested in DNPP from three elevations, 640 m, 675 m, and 725 m between August 29 and September 1, 2008 by Dr. C. Roland of the National Park Service. The cones were stored at a temperature of −26°C. In December 2009, spruce seeds were placed onto moist filter papers in petri dishes and stratified at 4°C for more than sixty days. In March 2010, seeds were moved into a growth chamber at a temperature of 21°C and given 14 hours of light each day to trigger germination (germination rate 38.0%). Each germinated seed was planted in soil (greenhouse standard media) in a pot measuring 4 cm diameter and a depth of 20 cm. Seedlings were watered with distilled water every other day for 10 days, then 10–30 - 20 (Nitrogen 10%, Phosphorus 30%, and Potassium 20%) fertilizer was applied at 50 mg/L N for the first 10 days, followed by 100 ppm N fertilizer for a week and 200 ppm N for about 45 days. Of 320 planted seeds, 53.4% survived (171 seedlings). Seedlings were assigned to treatments (as described below; Table 3) so that the seedlings with the same origin/similar size were evenly spread throughout treatments. At α = 0.05 significance level, mean height, number of needles, and the longest needle in each treatment group when planted were not significantly different among treatments (F _(11,159)_ = 1.702, P = 0.077, F _(11,159)_ = 1.210, P = 0.284, F _(11,159)_ = 1.690, P = 0.079, respectively).

**Table 3:** **Treatments, seed origin, original size and number of survivals of seedlings in September 2011**

**Habitat**	**Temperature**	**Neighbors**	**Site#**	**n**	**Origin 1**	**Origin 8**	**Origin 9**	**Height (cm)**	**#Needles**	**Length (cm)**	**Dead**	**Survived n**
Above	Control	Control	a	15	3	0	12	4.56 (1.60)	136.6 (53.1)	1.47 (0.20)	4	11
treeline	Control	Removal	b	14	3	0	11	4.49 (1.52	150.0 (61.0)	1.54 (0.19)	0	14
(1169 m)	High	Control	c	14	3	0	11	4.00 (1.43)	118.4(53.5)	1.49 (0.19)	0	14
	High	Removal	d	14	3	0	11	4.17 (1.85)	113.1 (46.9)	1.46 (0.12)	0	14
	**Above site total/average**			**57**				**4.31 (1.60)**	**129.5 (53.6)**	**1.49 (0.18)**	**4**	**53**
Near	Control	Control	e	15	3	0	12	4.02 (1.07)	162.2 (64.5)	1.55 0.23)	0	15
treeline	Control	Removal	f	14	3	0	11	4.86 (1.53)	173.9 (82.1)	1.61 (0.21)	0	14
(680 m)	High	Control	g	14	3	0	11	4.77 (1.13)	142.2 (43.2)	1.59 (0.21)	1	13
	High	Removal	h	14	3	1	10	5.69 (0.84)	146.6 (42.2)	1.59 (0.17)	0	14
	**Near site total/average**			**57**				**4.84 (1.14)**	**156.23 (58.0)**	**1.59 (0.21)**	**1**	**56**
Below	Control	Control	k	15	3	1	11	4.13 (1.30)	139.5 (54.3)	1.39 (0.16)	2	13
treeline	Control	Removal	l	14	3	1	10	4.36 (1.27)	156.9 (70.6)	1.47 (0.21)	0	14
(617 m)	High	Control	i	14	3	1	10	4.94 (1.88)	155.3 (78.8)	1.49 (0.19)	8	6
	High	Removal	j	14	3	1	10	4.96 (1.44)	162.4 (67.2)	1.55 (0.20)	0	14
	**Below site total/average**			**57**				**4.60 (1.47)**	**153.5 (67.7)**	**1.48 (0.19)**	**10**	**47**
	**Total/Average**			**171**				**4.58 (1.41)**	**146.4 (59.8)**	**1.52 (0.19)**	**15**	**156**

A total of 12 treatments were tested on 171 white spruce seedlings. After two summers, 156 (or 91%) of seedlings survived. In the “High” treatment, temperature was increased by small greenhouses. Above ground neighboring plants were removed in “Removal” plots. “Origin” is where the cones of the seeds were collected. “Origin 1” = 675 m, “Origin 8” = 725 m, “Origin 9” = 640 m. Mean “Height,” number of needles “#Needles,” and length of the longest needles “Length” (+SD) were measured at the time of planting. “Dead” and “Survived n” were recorded in September 2011.

### Treatments

The spruce seedlings were planted at the study area between June 2 and 11, 2010, under 12 treatments, consisting of 3 habitat types, 2 temperature levels, and 2 competition levels (Table 3). Three habitat types were: 1) above treeline on an exposed ridge with alpine tundra vegetation; 2) near treeline in shrub tundra; and 3) below treeline in a mature *P. glauca* forest (control); as described above.

**Figure 3:**
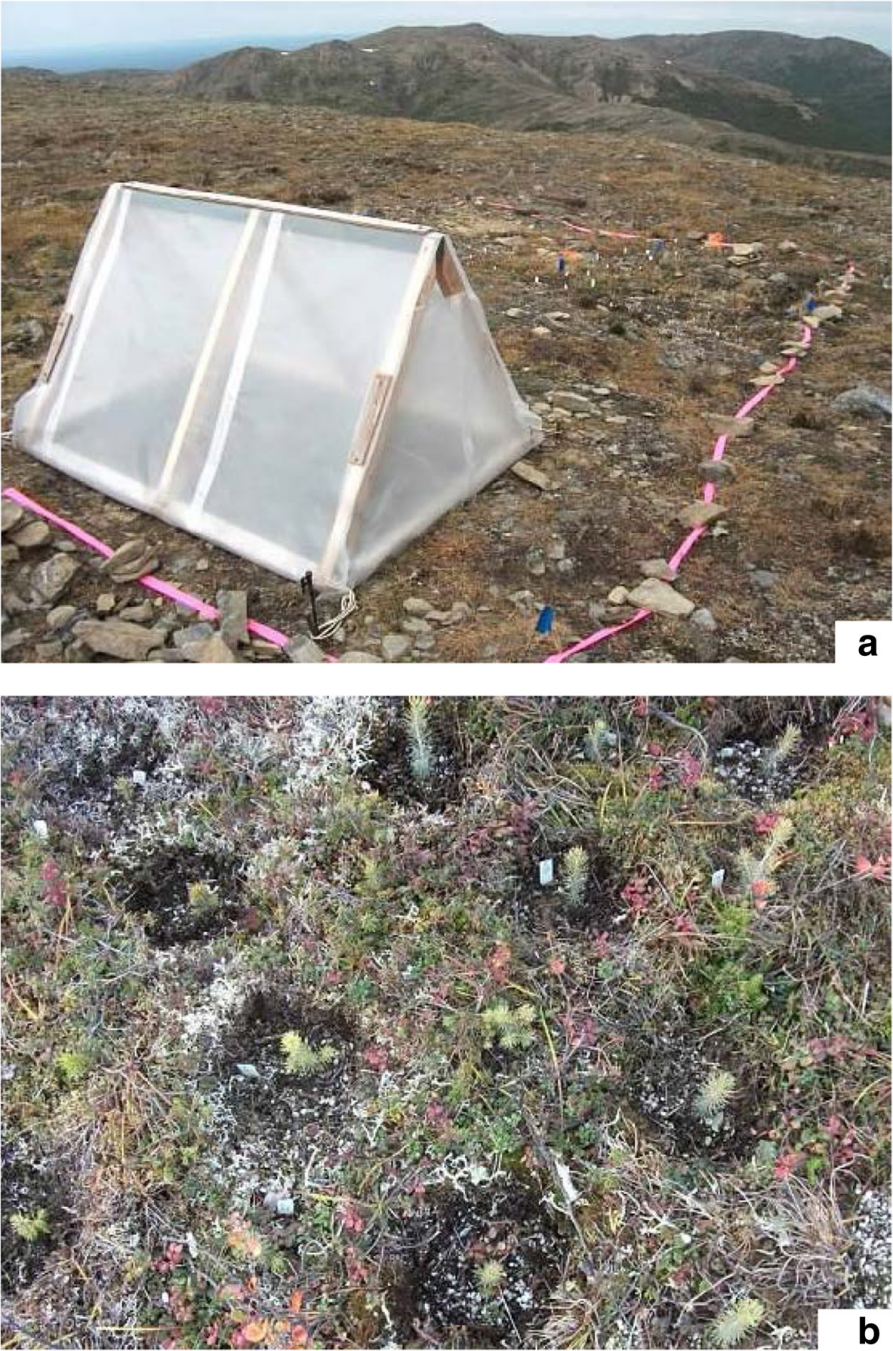
**Treatments. (a)** Greenhouse (elevated temperatures) and outside (ambient temperatures = control) sites above the treeline sites. **(b)** Removal (presence of competition) and non-removal (absence of competition = control) sites.

Two plots per site were established whose temperature levels were either 1) elevated temperatures to simulate climate change or 2) ambient temperatures (control). In order to passively increase air temperatures, small greenhouses were used in each habitat type during the summer seasons. Greenhouses were put into place in an early June and disassembled in late August of each year. An A-frame greenhouse (Figure 3a) constructed of 140 cm × 120 cm rectangular wooden frames with a height of 85 cm was covered with transparent 0.15 mm (0.6 mil) plastic sheeting. Top parts of the front and back walls were loosely opened for air ventilation. Through two growing seasons, air temperatures and relative humidity (RH, only in 2011) were recorded using temperature loggers (iButtons, DS1921, DS1923; Maxim Integrated, San Jose, CA). One logger was placed at the center of a greenhouse or an outside site for taking air measurements. Another logger for soil temperatures was buried just below the air logger.

In previous studies, no effect of greenhouses on soil moisture was reported (Bret‐Harte et al. 2002, Rixen and Mulder 2009) despite a lack of precipitation inside. The effect of winds could have been minimized by a greenhouse during three months of the summer growing season, but not during the majority of the year (9 months) after the greenhouse was disassembled in September, including winter when physical damage of seedlings due to wind was mainly expected.

Two levels of competition (Figure 3b) were 1) the absence of competition - removal of neighboring plants and 2) the presence of competition - neighboring plants present (control). Each seedling was planted at the center of a 15–18 cm diameter plot (Choler et al. 2001), which was large enough that seedlings whose neighbors were removed would not be shaded by surrounding plants, with 2–5 cm of buffer zones between seedlings. One seedling was planted with naturally occurring neighbors in the plot, while the next seedling was planted in a plot where aboveground biomass of neighboring species was removed by clipping. Belowground biomass was not removed, but the border soil between the plot and outside vegetation was cut to a maximum depth of 25 cm with a knife to minimize belowground competition. This condition was maintained by clipping the regrowth of neighboring plants in June and September each year. If competition were occurring, seedlings would grow better without neighbors. If facilitation were occurring, seedlings would grow better with neighbors.

Our experiment was conducted at a remote location with limited access, which limited what we could do. The most important limitation was that we could only have one greenhouse and one outside plot per site, in which 14–15 replicated seedlings were planted. This design limits our ability to extrapolate across the landscape, even though we established our plots on representative locations for each habitat type. Soil moisture was not statistically different between greenhouse and control plots in the same site where we could measure it (Table 1; t test, P = 0.45 above treeline, P = 0.49 near treeline). Given the constraints of doing research in a private inholding in the National Park, and the need to conduct measurements over a short period of time to be biologically reasonable, we felt this design was the best compromise.

### Data collection and analyses

After two summers and one winter at the end of the growing season of 2011, data were collected from 156 surviving seedlings (survival was 91%). Analyses included non-destructive size measurements, maximum gross photosynthetic rates, and concentrations of foliar nitrogen (N), carbon (C), and the natural abundances of ^15^N, and ^13^C (expressed as δ^15^N and δ^13^C, respectively) from a subset of samples.

Size data was obtained between August 27 and 31, consisting of height, number of needles and the longest leaf length of each seedling. Based on size measurements in May 2011, relative growth rates (RGR) that were used for statistical analyses were calculated for a comparison as follows:

$$ \mathrm{R}\mathrm{G}{\mathrm{R}}_{\mathrm{size}}\kern0.5em =\kern0.5em \left( \ln\ \mathrm{S}\mathrm{i}\mathrm{z}{\mathrm{e}}_{\mathrm{final}}\kern0.5em \hbox{-} \kern0.5em  \ln\ \mathrm{S}\mathrm{i}\mathrm{z}{\mathrm{e}}_{\mathrm{initial}}\right)/\ \left(\mathrm{d}\mathrm{a}{\mathrm{y}}_{\mathrm{final}}\kern0.5em \hbox{-} \kern0.5em \mathrm{d}\mathrm{a}{\mathrm{y}}_{\mathrm{initial}}\right) \dots .\ \mathrm{Durations}\kern0.75em =\kern0.5em 90 \sim 93\ \mathrm{da}\mathrm{ys} $$

Maximum photosynthetic capacity of seedlings was measured between 11:50 am and 5:25 pm from August 28 to September 3. For the seedlings in a greenhouse, measurements were taken on the day when the greenhouse sheeting was removed. Net photosynthesis and dark respiration rates were measured from all but one seedling (one failed, n = 155) using a LiCor 6400XT portable photosynthesis system (LiCor, Lincoln, NE, USA) under the same CO_2_ concentration of 400 ppm. Maximum net photosynthesis rates were recorded every 2 seconds for one minute (30 records) after placing a sample chamber over a seedling 2 cm from its top and exposing the seedling to a light intensity of 1500 μmol m^−2^ s^−1^ (full sunlight level) for two minutes. This shoot included a mixture of new and old needles. Just after photosynthesis was recorded, the light was turned off (0 μmol m^−2^ s^−1^ light intensity). After one minute of seedling acclimation to darkness, 30 dark respiration records were measured with the same time interval. Each set of 30 records was averaged and used to calculate an average gross photosynthesis rate per seedling as follows:

$$ \mathrm{Gross}\ \mathrm{photosynthesis} = \mathrm{N}\mathrm{e}\mathrm{t}\ \mathrm{photosynthesis}\ \left(\mathrm{a}\ \mathrm{positive}\ \mathrm{value}\right)\ \hbox{--}\ \mathrm{Dark}\ \mathrm{respiration}\ \left(\mathrm{a}\ \mathrm{negative}\ \mathrm{value}\right) $$

We assume that measurements in the dark represent respiration in the light, although we acknowledge that gross photosynthesis defined in this way possibly overestimates respiration because of the Kok effect, the inhibition of respiration in the light (Kok 1948, Heskel et al. 2013). However, very little difference between day and night time respiration of black spruce seedlings was seen previously (Way and Sage 2008).

We wanted to know whether the reduction of the light intensity by the greenhouse sheeting was substantial or negligible for seedlings’ photosynthetic performance. In order to estimate the maximum capacity of photosynthetic performance, a light response curve was created in March 2011, using 9, two-month old seedlings raised in a greenhouse from the same set of cones originating from Denali, with the same protocol stated previously. In addition, in the field, light levels inside and outside the greenhouses were measured using a light sensor on the LiCor 6400XT photosynthesis system. The sensor was placed facing directly to the sun light and data were recorded every 2 seconds for one minute. Data were obtained both sunny and overcasting days, obtaining 2–4 data per either inside or outside a greenhouse, then averaged.

Leaf area of each seedling was estimated by establishing a relationship between the total length of needles held in the sample chamber and their area. The needles used by photosynthesis measurements could not be harvested because this would damage the seedlings. Instead, needles of a-month-old seedlings that were from the same sets of cones but not planted in the field, were harvested prior to the field experiment. Leaf length and area of all harvested needles were measured and a linear regression between leaf area and leaf length was constructed:

$$ \mathrm{Leaf}\ \mathrm{area}\ \left({\mathrm{cm}}^2\right)\kern0.75em =\kern0.75em 0.0781\ *L*N $$

Where *L* is the average length of the needles and *N* is number of needles used. In the field, the number of needles 2 cm from the top of each seedling was counted, and the lengths of four representative needles were measured. Area was estimated from the length of those needles.

In order to investigate the relationship of foliar N and C with photosynthesis rates, 10–15 needles were collected from a subset of seedlings (n = 5 per treatment, 59 total due to failure of a photosynthetic rate) after each size measurements. Stable isotope fractionation in δ^15^N and δ^13^C was focused to explain plants’ dependency in nitrogen on mycorrhizal association (δ^15^N) and plants’ drought stress level (δ^13^C). Samples were dried at 60°C and ground. Concentration of N, C, δ^15^N and δ^13^C were determined by continuous flow isotope ratio mass spectrometry using a Costech ECS4010 Elemental Analyzer (Costech Analytical Technologies Inc.,Valencia, CA) interfaced to a Finnigan Delta plus XP isotope ratio mass spectrometer via Conflo III interface (Thermo Fisher Scientific Inc., Waltham, MA) at the Alaska Stable Isotope Facility at UAF.

Other environmental data were obtained either in the fall of 2010 or 2011. Three soil samples per site (<5 m from the plots) were collected by cutting soil with a knife and measured the volume. Each sample was dried at 105°C over 3 days and weighed to calculate a bulk density and water content. Another sample per site was collected for CN, pH, and texture tests. The percentage of C, N contents of soil was analyzed at the Forest Soils Laboratory at the UAF School of Natural Resources and Extension. Soil pH was tested using a pH test indicator (General Hydroponics-USA, Sebastopol, CA, USA). Texture was defined by Bouyoucos Hydrometer Method. In addition to sampling, soil volumetric water content was measured using a Hydrosense soil water measurement system (Cambell Scientific Inc., North Logan, UT, USA).

Data on size measurements, gross photosynthesis rates, and foliar CN data were analyzed using three-way ANOVA (general linear model (GLM) with habitat, temperature and competition as main effects and all possible interactions). Post-hoc tests (Tukey’s HSD) were used to interpret significant effects. A subset of the photosynthesis data was also analyzed by ANCOVA. Either N concentration or δ^13^C was used as a separate covariate in order to assess whether foliar %N or fractionation of δ^13^C explained the differences in photosynthetic performance. Data were tested for homogeneity of variance before the analysis using four tests (O’Brien, Brown-Forsythe, Bartlett and Levene); if necessary, data were transformed using ranking, weighted standardization, or log transformation as indicated in Table 2. When interaction terms were significant, data were further analyzed using one-way ANOVA.

## Additional file

Additional file 1: Figure: S1. Light response curvefrom two-month old seedlings (n = 9). Error bars show one standard error. **Figure S2.** Relationship between %N and δ^15^N of subsampled needles. δ^15^N = − (0.5225932 foliar %N) + 3.4072357. (n = 59, r^2^ = 0.02, P = 0.2588).

## References

[CR1] Anderson LL, Hu FS, Nelson DM, Petit RJ, Paige KN (2006). Ice-age endurance: DNA evidence of a white spruce refugium in Alaska. P Natl Acad Sci USA.

[CR2] Barber VA, Juday GP, Finney BP (2000). Reduced growth of Alaskan white spruce in the twentieth century from temperature-induced drought stress. Nature.

[CR3] Bret‐Harte MS, Shaver GR, Chapin FS (2002). Primary and secondary stem growth in arctic shrubs: implications for community response to environmental change. J Ecol.

[CR4] Bret-Harte MS, Mack MC, Goldsmith GR, Sloan DB, DeMarco J, Shaver GR, Ray PM, Biesinger Z, Chapin FS (2008). Plant functional types do not predict biomass responses to removal and fertilization in Alaskan tussock tundra. J Ecol.

[CR5] Callaway RM (1998). Competition and facilitation on elevation gradients in subalpine forests of the northern Rocky Mountains, USA. Oikos.

[CR6] Callaway RM, Brooker RW, Choler P, Kikvidze Z, Lortie CJ, Michalet R, Paolini L, Pugnaire FI, Newingham B, Achehoug ET, Armas C, Kikodze D, Cook BJ (2002). Positive interactions among alpine plants increase with stress. Nature.

[CR7] Carter RE (1992) Diagnosis and interpretation of forest stand nutrient status. In: Chappell HN, Weetman GF, Miller RE (eds) Forest fertilization: sustaining and improving nutrition and growth of western forests. College of Forest Resources Contribution #73. University of Washington, Seattle, pp 90–97

[CR8] Cavieres LA, Arroyo MTK, Molina-Montenegro M, Torres C, Peñaloza A (2002). Nurse effect of *Bolax gummifera* (*Apiaceae*) cushion plants in the alpine vegetation of the Chilean Patagonian Andes. J Veg Sci.

[CR9] Choler P, Michalet R, Callaway RM (2001). Facilitation and competition on gradients in alpine plant communities. Ecology.

[CR10] Cortini F, Comeau PG, Bokalo M (2012). Trembling aspen competition and climate effects on white spruce growth in boreal mixtures of Western Canada. Forest Ecol Manag.

[CR11] Cortini F, Comeau PG (2008). Evaluation of competitive effects of green alder, willow and other tall shrubs on white spruce and lodgepole pine in Northern Alberta. Forest Ecol Manag.

[CR12] Dalen L, Hofgaard A (2005). Differential regional tree line dynamics in the Scandes Mountains. Arct Antarct Alp Res.

[CR13] Danby RK, Hik DS (2007). Responses of white spruce (*Picea glauca*) to experimental warming at a subarctic alpine treeline. Glob Change Biol.

[CR14] de Lafontaine G, Turgeon J, Payette S (2010). Phylogeography of white spruce (*Picea glauca*) in eastern North America reveals contrasting ecological trajectories. J Biogeogr.

[CR15] Díaz S, Hodgson JG, Thompson K, Cabido M, Cornelissen JHC, Jalili A, Montserrat-Martí G, Grime JP, Zarrinkamar F, Asri Y, Band SR, Basconcelo S, Castro-Díez P, Funes G, Hamzehee B, Khoshnevi M, Pérez-Harguindeguy N, Pérez-Rontomé MC, Shirvany FA, Vendramini F, Yazdani S, Abbas-Azimi R, Bogaard A, Boustani S, Charles M, Dehghan M, de Torres-Espuny L, Falczuk V, Guerrero-Campo J, Hynd A, Jones G, Kowsary E, Kazemi- Saeed F, Maestro-Martínez M, Romo-Díez A, Shaw S, Siavash B, Villar-Salvador P, Zak MR (2004). The plant traits that drive ecosystems: evidence from three continents. J Veg Sci.

[CR16] Gervais BR, MacDonald GM (2000). A 403-year record of July temperatures and treeline dynamics of Pinus sylvestris from the Kola Peninsula, northwest Russia. Arct Antarct Alp Res.

[CR17] Goldstein G, Brubaker GL, Hinckley T (1985). Water relations of white spruce (*Picea glauca* (Moench) Voss) at treeline in north-central Alaska. Can J For Res.

[CR18] Grime JP (2001). Plant strategies, vegetation processes, and ecosystem properties.

[CR19] Grime J, Thompson K, Hunt R, Hodgson JG, Cornelissen JHC, Rorison IH, Hendry GAF, Ashenden TW, Askew AP, Band SR, Booth RE, Bossard CC, Campbell BD, Cooper JEL, Davison AW, Gupta PL, Hall W, Hand DW, Hannah MA, Hillier SH, Hodkinson DJ, Jalili A, Liu Z, Mackey JML, Matthews N, Mowforth MA, Neal AM, Reader RJ, Reiling K, Ross-Fraser W, Spencer RE, Sutton F, Tasker DE, Thorpe PC, Whitehouse J (1997). Integrated screening validates primary axes of specialisation in plants. Oikos.

[CR20] Grossnickle SC (2000). Ecophysiology of Northern spruce species: the performance of planted seedlings.

[CR21] Hamm J (2007) Recent tree line advance and the influence of shrub and tundra communities on white spruce (*Picea glauca*) establishment in Denali National Park, Alaska. Master thesis. Department of Environmental Studies, Antioch New England Graduate School, Antioch University, USA

[CR22] Heskel MA, Atkin OK, Turnbull MH, Griffin KL (2013). Bringing the Kok effect to light: a review on the integration of daytime respiration and net ecosystem exchange. Ecosphere.

[CR23] Hobbie SE, Chapin FS (1998). An experimental test of limits to tree establishment in Arctic tundra. J Ecol.

[CR24] Hobbie JE, Hobbie EA, Drossman H, Conte M, Weber JC, Shamhart J, Weinrobe M (2009). Mycorrhizal fungi supply nitrogen to host plants in Arctic tundra and boreal forest: ^15^ N is the key signal. Can J Microbiol.

[CR25] IPCC (2008) Synthesis report. In: Core Writing Team, Pachauri RK, Reisinger A (eds) Climate change 2007: Synthesis report. Contribution of working groups I, II and III to the fourth assessment report of the Intergovernmental Panel on Climate Change. Intergovernmental Panel on Climate Change, Geneva

[CR26] Juday GP, Barber V, Duffy P, Linderholm H, Rupp TS, Sparrow S, Vaganov E, Yarie J (2005) Forests, land management,and agriculture. In: ACIA (ed) Arctic Climate Impact Assessment. Cambridge University Press, New York

[CR27] Karl TR, Melillo JM, Peterson TC (2009). Regional climate impact: Alaska. Global climate change impacts in the United States.

[CR28] Kok B (1948). A critical consideration of the quantum yield of Chlorella-photosynthesis. Enzymologia.

[CR29] Körner C (2012). Treelines will be understood once the functional difference between a tree and a shrub is. Ambio.

[CR30] Kullman L (1991). Structural change in a subalpine birch woodland in north Sweden during the past century. J Biogeogr.

[CR31] Litton CM, Giardina CP (2008). Below-ground carbon flux and partitioning: global patterns and response to temperature. Funct Eco.

[CR32] Livingston NJ, Guy RD, Sun ZJ, Ethier GJ (1999). The effects of nitrogen stress on the stable carbon isotope composition, productivity and water use efficiency of white spruce (*Picea glauca* (Moench) Voss) seedlings. Plant Cell Environ.

[CR33] Lloyd AH, Fastie CL (2003). Recent changes in treeline forest distribution and structure in interior Alaska. Ecoscience.

[CR34] Man R, Lieffers VJ (1997). Seasonal photosynthetic responses to light and temperature in white spruce (*Picea glauca*) seedlings planted under an aspen (*Populus tremuloides*) canopy and in the open. Tree Physiol.

[CR35] Nienstaedt H, Zasada JC (1990) *Picea glauca*. In: Burns RM, Honkala BH (eds) Silvics of North America: 1. Conifers; 2. Hardwoods. Agriculture Handbook 654. U.S. Department of Agriculture, Forest Service, Washington, DC

[CR36] Rixen C, Mulder CP (2009). Species removal and experimental warming in a subarctic tundra plant community. Oecologia.

[CR37] Roland CA, Schmidt JH, Johnstone JF (2013) Climate sensitivity of reproduction in a mast- seeding boreal conifer across its distributional range from lowland to treeline forests. Oecologia: 1–13 doi 10.1007/s00442-013-2821-6 10.1007/s00442-013-2821-6 24213628

[CR38] Slot M, Wirth C, Schumacher J, Mohren GM, Shibistova O, Lloyd J, Ensminger I (2005). Regeneration patterns in boreal scots pine glades linked to cold-induced photoinhibition. Tree Physiol.

[CR39] Stitt M, Schulze D (1994). Does Rubisco control the rate of photosynthesis and plant growth? An exercise in molecular ecophysiology. Plant Cell Environ.

[CR40] Stueve KM, Isaacs RE, Tyrrell LE, Densmore RV (2011). Spatial variability of biotic and abiotic tree establishment constraints across a treeline ecotone in the Alaska Range. Ecology.

[CR41] Sullivan PF, Ellison SB, McNown RW, Brownlee AH, Sveinbjörnsson B (*In press*) Evidence of soil nutrient availability as the proximate constraint on growth of treeline trees in northwest Alaska. Ecology. 10.1890/14-0626.1 26236868

[CR42] Sturm M, Racine C, Tape K (2001). Climate change: increasing shrub abundance in the Arctic. Nature.

[CR43] Sveinbjörnsson B (2000). North American and European treelines: external forces and internal processes controlling position. AMBIO: A J Hum Environ.

[CR44] Szeicz JM, MacDonald GM (1995). Recent white spruce dynamics at the subarctic alpine treeline of northwestern Canada. J Ecol.

[CR45] Tape K, Sturm M, Racine C (2006). The evidence for shrub expansion in Northern Alaska and the Pan-Arctic. Glob Change Biol.

[CR46] Tilman D (1988). Plant strategies and the structure and dynamics of plant communities.

[CR47] USDA, ARS, National Genetic Resources Program. Germplasm Resources Information Network - (GRIN) [Online Database].National Germplasm Resources Laboratory, Beltsville, Maryland. URL: http://www.ars-grin.gov/cgi-bin/npgs/html/taxon.pl?433404 (25 October 2014)

[CR48] Waring RH (1987). Characteristics of trees predisposed to die. Stress causes distinctive changes in photosynthate allocation. BioScience.

[CR49] Way DA, Sage RF (2008). Thermal acclimation of photosynthesis in black spruce [*Picea mariana* (Mill.) BSP]. Plant Cell Environ.

[CR50] Wilmking M, Juday GP, Barber V, Zald HSJ (2004). Recent climate warming forces contrasting growth responses of white spruce at treeline in Alaska through temperature thresholds. Glob Change Biol.

[CR51] Yin HJ, Liu Q, Lai T (2008). Warming effects on growth and physiology in the seedlings of the two conifers *Picea asperata* and *Abies faxoniana* under two contrasting light conditions. Ecol Res.

[CR52] Zhang SR, Dang QL (2007). Interactive effects of soil temperature and CO_2_ on morphological and biomass traits in seedlings of four boreal tree species. Forest Sci.

