# Supplementary material for: Warming and neighbor removal affect white spruce seedling growth differently above and below treeline
Source: Springerplus. 2015 Feb 13;4:79. doi: 10.1186/s40064-015-0833-x (PMC4339320; doi:10.1186/s40064-015-0833-x)
Supplement: Additional file 1: Figure: S1. — Light response curvefrom two-month old seedlings (n = 9). Error bars show one standard error. Figure S2. Relationship between %N and δ15N of subsampled needles. δ15N = − (0.5225932 foliar %N) + 3.4072357. (n = 59, r2 = 0.02, P = 0.2588). [file 40064_2015_833_MOESM1_ESM.docx]

**Title:** Warming and neighbor removal affect white spruce seedling growth differently above and below treeline

**Authors:** Kyoko Okano, M. Syndonia Bret-Harte

**Supplemental Figure Legends**

**Fig. S1** Light response curve obtained from two-month old seedlings (n = 9). Error bars show one standard error

**Fig. S2** Relationship between %N and δ^15^N of subsampled needles. δ^15^N = – (0.5225932 foliar %N) + 3.4072357. (n = 59, *r*^2^ = 0.02, *P* = 0.2588)


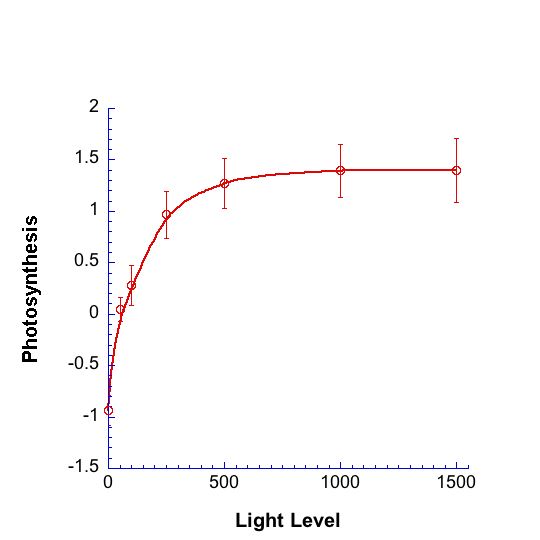


**(μmol CO_2_ m^-2^ s^-1^)**

**(μmol m^-2^ s^-1^)**

**Fig. S1**


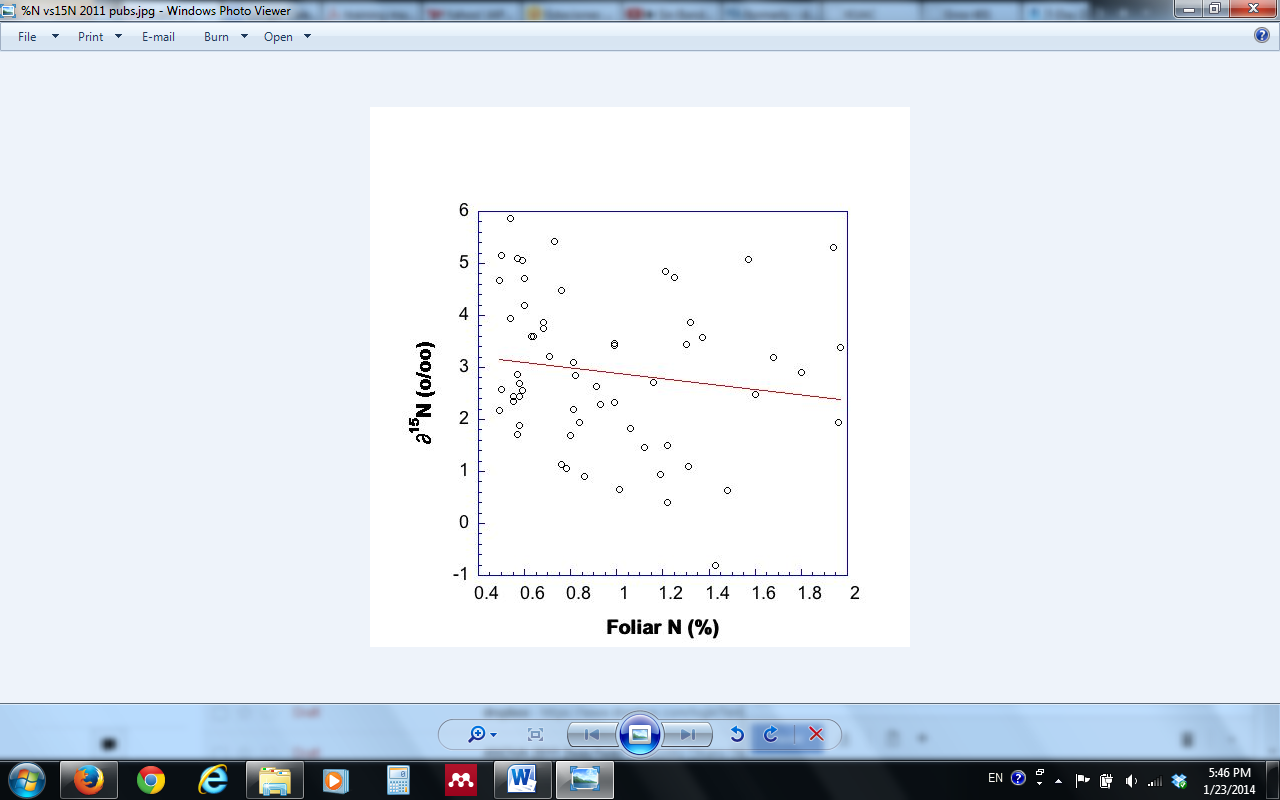


**Fig. S2**
